# Examining the Relationship between Salivary Amylase Level, Head Trauma Severity and CT Scan Results in Patients with Isolated Mild Head Trauma 

**DOI:** 10.30476/BEAT.2022.94151.1330

**Published:** 2022-04

**Authors:** Mohsen Ebrahimi, Behrang Rezvani Kakhki, Baharak Davoudpour, Zahra Abbasi Shaye, Hossein Zakeri, Seyed Mohammad Mousavi, Sayyed Majid Sadrzadeh, Seyed Aliakbar Shamsian, Azadeh Mahmoudi Gharaee

**Affiliations:** 1 *Department of Emergency Medicine, Faculty of Medicine, Mashhad University of Medical Sciences, Mashhad, Iran*; 2 *Clinical Research Development Center, Faculty of Medicine, Mashhad University of Medical Sciences, Mashhad, Iran*; 3 *Department of Parasitology and Mycology, Faculty of Medicine, Mashhad University of Medical Sciences, Mashhad, Iran*; 4 *Emergency Physician Specialist, Talghani Hospital, Mashhad University of Medical Sciences, Mashhad, Iran*

**Keywords:** Salivary alpha-amylase, Computed tomography (CT scan), Head trauma, Glascow coma scale (GCS)

## Abstract

**Objective:**

To investigate the relationship between salivary amylase level and computed tomoraphy (CT scan) findings in patients with isolated mild traumatic Brain Injury (mTBI) referred to the emergency department of Shahid Hasheminejad Hospital.

**Methods:**

Patients with isolated mTBI and indication for brain CT scan who referred to the trauma center of Shahid Hasheminejad Hospital, Mashhad, Iran in 2019 were included in a cross-sectional study. In the initial examination, the patient’s level of consciousness was measured using the Glasgow Coma Scale (GCS), and saliva samples were taken at the emergency department to determine the level of salivary amylase. A brain CT scan was performed for all patients. Age, gender, cause of trauma, the trauma severity and CT scan results were recorded. Statistical analysis was performed on the data.

**Results:**

One-hundred fifty patients were enrolled in this study (men=101, women=49). The trauma causes were included accidents (n=88; 58%), falls (n=37; 25%) and miscellaneous factors (e.g., quarrels; n=25; 17%). GCS was 15 in 142 patients and 14 in the rest. In all patients, the trauma severity was mild to high risk (Minor). CT scan results unfolded pathology in 10 cases (7%), while the residues (93%) had normal CT scans with no pathological evidence. Salivary amylase level in the patients’ saliva samples was between 137 to 8000 units per liter. Using the t-test to evaluate the relationship between salivary amylase levels and CT scan results uncovered a significant relationship. Spearman correlation revealed no significant relationship between the amylase and GCS levels.

**Conclusion:**

Data statistical analysis from 150 patients with isolated head trauma manifested that salivary amylase levels were significantly higher in the patients with pathological findings on CT scans. However, no significant relationship was found between salivary amylase level and age, gender, cause of trauma, and level of consciousness.

## Introduction

Traumatic brain injury (TBI) is a leading cause of death and permanent disabilities in young adults worldwide, with an increasing prevalence in the elderly population. Despite recent advances in intensive care, the rate of mortality and disability due to this injury remain high [1]. Immediate diagnosis of the injury severity and predicting its outcome can improve patients’ care and, clarifies the pros and cons of early treatment options [2]. Survivors of TBI experience limitations in daily activities, using various devices, social adjustment, and economic independence. About 43% of patients discharged from hospitals with this diagnosis develop long-term disabilities. A history of traumatic brain injury is strongly associated with subsequent neurological disorders, including Alzheimer’s and Parkinson’s diseases, which in turn cause more disability for the patients. Treatment, social protection, disabilities, and other outcomes of TBI damages impose huge costs on countries each year [3]. On the other hand, these costs increase since the majority of patients are young [4]. In a study in Rasht, most TBI patients were students in the age range of 15-24 years of old [5]. In a study conducted in Egypt in 2017, the relationship between injury severity and patients’ survival with serum levels of amylase, lipase, and Gamm Glutamyltranserase (GGT) was investigated. In this study, the patients’ blood samples were taken twice [1], at the time of admission and [2] 24 hours later. The serum level of these markers in both blood sampling sessions was significantly lower in the patients with GCS as well as those who did not survive during hospitalization. Also, these three variables were suggested as predictors of the final outcome in the TBI patients [6]. In another study, 60 patients with severe TBI and GCS less than 10 were compared with 14 patients with traumas to other organs except for the head. The serum amylase levels were measured on days 0, 2, 4, 7, and 14 days after the trauma. The serum amylase levels in the first group were significantly higher, which introduces the head trauma as the possible cause of increased serum amylase [7]. Due to the high prevalence of head injuries in accidents, lack of CT scan equipment in many emergency departments, and time shortage to deliver TBI patients to other equipped centers, using available markers to assess the severity of brain damage with acceptable accuracy seems necessary, and it also helps in estimating TBI patients’ prognosis. The study of serum biomarkers has played a significant role in this. Many studies have used coagulation tests [8], neurofilament [2], and serum lipase and amylase enzymes [2, 7, 9] to assess the severity of TBIs. Regarding limited studies on TBIs in Iran on the one hand and the high rate of TBIs due to the demographic context and traffic safety, on the other hand, the present study was performed to investigate the relationship between salivary amylase and the severity of brain trauma injuries and CT scan results in patients with isolated head trauma referred to the emergency department of Shahid Hasheminejad hospital.

## Materials and Methods

In this cross-sectional descriptive study, patients with isolated head trauma and indicated for brain CT scan who referred to the trauma center of Shahid Hasheminejad hospital in Mashhad were selected by the available sampling method. The sample size was calculated at 157 individuals based on the suggestion of Vitale GC *et al*., [7] as well as the estimation of the hyperamylasmia ratio due to the assumptions of the above formula (alpha: 0.05, beta: 0.1, p: 38%). However, it was increased to 190 individuals to make sure of accuracy and validity. Inclusion criteria was mTBI, defined as GCS≥13, and in case of any pathology in Brain CT scan findings, patients with subclinical findings in brain CT scan including contusions, tiny subarachnoid or intraparenchymal hemorrhages, subdural and epidural collections, edema, and minor skull fractures were included [10]. 

 After explaining the study plan to the patients, verbal consent was taken. For the patients with a low level of consciousness, their legal guardians were asked to give informed consent. Confidentiality of the patients’ characteristics and findings was observed during all stages of the study. At the initial examination, the patients’ levels of consciousness were measured by the Glasgow Coma Scale (GCS). A nurse took a 1-2 cc of the saliva sample from each patient immediately after arrival at the emergency department. Saliva samples secreted without physical or chemical stimulation at the time of sampling were collected in a special tube. The sampling was performed only once at the patient’s arrival at the emergency department. The level of salivary amylase in all the samples was determined in the hospital laboratory. Then, the patients were taken Brain CT scans. 

The alpha-amylase activity was quantitatively measured by a DiaMetra salivary amylase assay kit. Amylase levels above 80 IU/mL were considered high and abnormal. Due to the lack of salivary amylase kit in the market at the time of patient recruitment and the impossibility of supplying it, and after the approval of the Vice-Chancellor for Research, the sample size was waived to 150 people.

Findings of the age, gender, cause and severity of the trauma, salivary amylase level, and CT scan outcomes were recorded. Trauma severity was divided into three levels based on GCS: minor with GCS of 13 to 15, moderate with GCS of 9 to 12, and severe with GCS of higher than 8. During the sampling we noticed that incidentally, all patients with GCS of ≤13 had concomitant multiple traumas. 

Pathological CT scan includes any bleeding (subarachnoid hemorrhage (SAH), Subdural hemorrhage (SDH), Extradural hematoma (EDH)) and minor fractures of the skull and its base. The data were analyzed by the SPSS Software-22 and described in terms of central descriptive tests and scattering (e.g., mean and standard deviation). Normal data distribution was confirmed by the central limit theorem. Independent t-test helped to compare the amylase level in the two gender groups and between the two groups with normal and abnormal CT scans. To determine the cause of trauma, an ANOVA test was performed. Pearson correlation test was used to examine the correlation between amylase levels and age. The relationship between the amylase level and GCS was examined by the Spearman correlation test. P≤0.05 was significant.

## Results

**Fig. 1:**
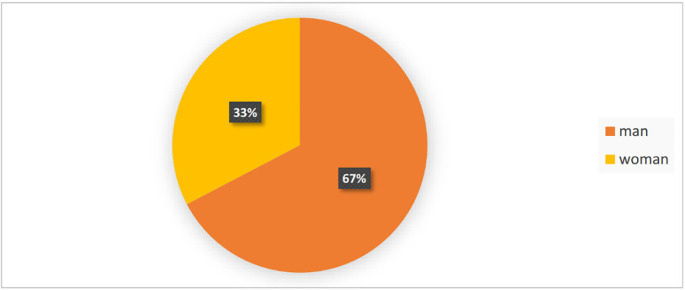
Relative frequency of patients’ gender

**Fig. 2:**
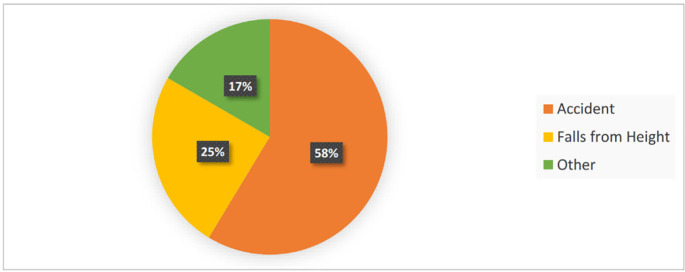
Relative frequency of causes of head trauma in patients

One-hundred fifty patients (men=101 (67%), and women=49 (33%)) were included in the study. Figure 1 shows the gender ratio of the patients. Causes of trauma were included accidents in 88 cases (58%), falls in 37 cases (25%), and miscellaneous factors, like quarrels, in 25 cases (17%). The percentage of different risk factors for head trauma is shown in Figure 2. The participants’ age means was 33.21±15.21 years (the oldest and youngest patients were 81 and 7, respectively). Information about the patients’ age by gender, cause of trauma, GCS level, and CT scan result is given in Table 1. Out of 150 patients, 142 patients had GCS=15 and only 8 patients had GCS=14. The trauma severity in all the patients was mild (minor); while based on the physicians’ decision was considered high risk, and required a CT scan (Table 1).

**Table 1:** Mean age (year) of patients by qualitative variables

		**N**	**Min (age)**	**Max (age)**	**Mean(age)**	**SD** ^a^
Sex	Male	101	8	80	31.94	13.53
	Female	49	7	74	35.82	18.7
Trauma mechanism	Accident	88	8	80	31.34	14.1
	Falling down	37	7	74	38.92	18.91
	Other	25	17	65	31.32	11.4
GCS^b^	14	8	9	67	39.62	19.53
	15	142	7	80	32.85	14.94
CT^c^ scan	Normal	140	7	80	33.37	15.32
	Abnormal	10	9	62	3.90	14.4
Total		150	7	80	33.21	33.21

^a^SD: Standard Deviation; ^b^GCS: Glascow Coma Scale; ^c^CT: Computed Tomography

**Fig. 3:**
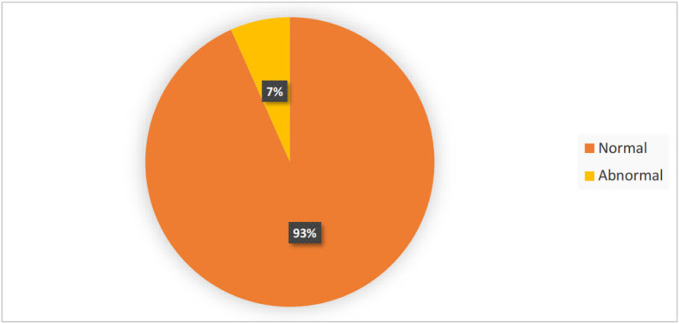
Relative frequency of CT scan results of patients

Pathological findings were observed in the CT scans of 10 patients. Figure 3 shows the ratio of CT scan results. Pathological CT scans in the patients included EDH, SAH, and sunken skull fractures (Table 2).

**Table 2:** Frequency of qualitative variables by each variable

		**Sex**				**Trauma mechanism**						**GCS** ^a^					**CT** ^b^ ** SCAN**			
		**Male**		**Female**		**Accident**		**Falling down**		**Other**		**14**			**15**		**Normal**		**Abnormal**	
		**N**	**%**	**N**	**%**	**N**	**%**	**N**	**%**	**N**	**%**	**N**		**%**	**N**	**%**	**N**	**%**	**N**	**%**
Sex	Male					57	8.64	22	5.59	22	88	6		75	95	9.66	93	4.66	8	80
	Female					31	2.35	15	5.4	3	12	2		25	47	1.33	47	6.33	2	20
Trauma mechanism	Accident	57	4.56	31	3.63							3		5.37	85	9.59	81	9.57	7	70
	Falling down	22	8.21	15	6.3							4		50	33	2.23	35	25	2	20
	Other	22	8.21	3	1.6							1		5.12	24	9.16	24	1.17	1	10
GCS^a^	14	6	9.5	2	1.4	3	4.3	4	8.1	1	4						2	4.1	6	60
	15	95	1.94	47	9.95	85	6.96	33	2.89	24	96						138	6.98	4	40
CT^b^ scan	Normal	93	1.92	47	9.95	81	92	35	6.94	24	96	2		25	138	2.97				
	Abnormal	8	9.7	2	1.4	7	8	2	4.5	1	4	6		75	4	8.2				

^a^GCS: Glascow Coma Sclae; ^b^CT: Computed Tomography

Salivary amylase level in the patients’ saliva samples was between 137 to 8000 units per liter, with the mean as 415.66±711.30 units per liter. Table 3 exhibits the mean, maximum, and minimum of salivary amylase levels in terms of the patients’ gender, cause of trauma, GCS level, and CT scan results.

**Table 3:** Average amounts of salivary amylase (Unit. Lit) by qualitative variables

		**N**	**Min**	**Max**	**Mean**	**SD** ^e^	***p*** ** value**
			**(Unit. Lit)**	**(Unit. Lit)**	**(Unit. Lit)**		
Sex	Male	101	137	8000	449.16	845.69	0.41^a^
	Female	49	138	1651	346.61	27.79	
Trauma mechanism	Accident	88	137	8000	432.75	862.9	0.83^b^
	Falling down	37	138	2052	354.92	385.89	
	Other	25	166	2286	445.40	464.71	
GCS^f^	14	8	138	8000	1258.38	2729.64	0.93^c^
	15	142	137	2286	368.18	349.55	
CT^g^ scan	Normal	140	137	2286	362.6	339.67	<0.001^d^
	Abnormal	10	184	8000	1166.10	2433.84	
Total		150	137	8000	415.66	711.3	

^a^T-test was used to examine the relationship between the two variables; ^b^ANOVA test was used to examine the relationship between the two variables; ^c^Spearman test was used to examine the relationship between the two variables;^ d^T-test was used to examine the relationship between the two variables; eSD: Standard Deviation; fGCS: Glascow Coma Scale; gCT: Computed Tomography

The t-test disclosed no significant relationship between amylase levels and gender (*p*=0.41). Also, no significant relationship was found between amylase levels and age (*p*=0.92), and between amylase levels and the cause of trauma (*p*=0.83), by the Pearson correlation and ANOVA tests, respectively. Amylase level and GCS were not significantly related by the Spearman correlation (*p*=0.93)

T-test was used to evaluate the relationship between salivary amylase levels and CT scan results, and a significant relationship was observed (*p*<0.001). In other words, significantly higher levels of salivary amylase were observed in the patients with abnormal CT scans. The P-value of the mentioned tests in examining the relationship between amylase levels and the variables (gender, age, cause of trauma, and CT scan results) is also shown in Table 3.

## Discussion

The results of the present study demonstrated that the majority of patients with isolated head trauma were young men. Additionally, only eight patients had decreased consciousness with GCS=14, and in 7% of the patients, the CT scan results were abnormal. The salivary amylase level was not significantly associated with the patients’ gender, age, and cause of trauma, but it was significantly related to the abnormal CT scan results. Bowman *et al*., [11] following the incidental finding of hyperamylasemia in a patient with isolated head trauma, examined ten similar patients with findings of intracranial hemorrhage on their CT scans. Six of these patients had hyperamylasemia, and high levels of amylase were observed in the CT scan of only one patient. Examining seven patients with isolated trauma to the jaw and face disclosed no high level of amylase. Analysis of Amylase isoenzymes revealed various origins for hyperamylasemia. The scholar concluded that the origin of hyperamylasmia due to isolated head trauma was probably a neurological process and not caused by a trauma to the salivary glands. The results of this study regarding the higher levels of amylase in patients with evidence of injury on CT scan are consistent with the findings of the present study. It also supports the exclusion of patients with anxiety disorders or disorders of the autonomic system from the sample of the present study. 

Detoldo *et al*., [12] examined the serum amylase levels of 51 children with severe TBIs and a GCS of less than 8. They found an increase in the amylase level in 29 children and observed that aging, increased intracranial pressure, and intracranial hemorrhage was associated with a delayed increase in serum amylase (after the first 24 hours). These factors were not evaluated in detail in our study that timing should be considered for further research. Abolnour *et al*., [6] reported the serum amylase, lipase, and GGT as the predictors of patient trauma outcomes, as well as a factor to investigate the association between patients’ death and head trauma in forensic evaluations. The findings of this study on the cause of head trauma are similar to that of ours. It also supports the increased pancreatic secretion in patients with more severe TBIs. In the study of Cheng Chia *et al*., [13] high levels of pancreatic enzymes were significantly associated with more mortality. The researchers recommended performing an abdominal CT scan to check for pancreatitis in patients with amylase higher than three times and lipase higher than five times of the normal range. Findings of this study on the relationship between amylase levels and the severity of TBIs advocate our findings. This difference can be explained by the fact that in the study of Cheng Chia *et al*., [14] the presence of gastrointestinal symptoms was an inclusion criterion, while in our study, patients with injuries in other organs except the head were excluded. It may be concluded that the application of amylase in patients with various traumas except isolated head trauma is very limited in diagnosing or predicting the severity of TBIs. Eventually, however, the patients with higher levels of pancreatic enzymes were more likely to die. Ewing-Cobbs *et al*., [13] investigated the symptoms of post-traumatic stress disorder (PTSD) in children and adolescents with trauma and reported that salivary amylase levels were higher in adolescents aged 13-15 with traumatic brain injuries compared to the adolescents with extra cranial injuries. The salivary amylase level was also linked to avoidance symptoms and emotional numbness in traumatized children and adolescents. 

Recent discoveries support our finding in terms of higher levels of salivary amylase in brain injuries compared to extra-cranial injuries. It also clears that examining the salivary amylase level may be useful for diagnosing and predicting post-traumatic anxiety symptoms. This relationship is important since post-traumatic stress disorder (PTSD) is responsible for a large part of disabilities. In another study by Bowman *et al*., [15] measuring the amylase level by both pancreatic and non-pancreatic isoenzymes did not improve the role of amylase in the evaluation of patients with abdominal traumas. This study suggested that the increased level of amylase enzyme in head trauma led to increased secretion of various amylase isoenzymes through a neural mechanism, rather than direct damage to amylase-secreting organs such as salivary glands.

The present study demonstrated that salivary amylase levels in patients with isolated head trauma were not significantly related to the age, gender, cause of trauma, and level of consciousness. It should be noted that in this study, accidentally, only patients with mild TBIs were assessed, and no significant relationship was observed between the level of consciousness (GCS) and salivary amylase; however, patients with moderate or severe TBIs should have Brain CT, salivary amylase cannot change this protocol, therefore, the results of salivary amylase levels are not significantly associated with the severity of mild TBIs in mild head trauma that is valuable. But, the salivary amylase level in patients with isolated head trauma was significantly associated with pathological findings in CT scans. The results of this study suggest that measuring the salivary amylase level could be a rapid, inexpensive, accessible, uncomplicated, minimally invasive, and reliable method to assess the risk of injury and the possibility of pathology on CT scan of patients with isolated head trauma.

## Limitations

This study had multiple limitations. First, to evaluate amylase level diagnostic accuracy in predicting any CT scan pathological findings, a low number of patients with abnormal CT scan among patients with GCS of 15, did not satisfy prerequisites of further statistical analysis. Therefore, we suggest further research in a higher number of samples to examine this hypothesis that could help decrease unnecessary CT scan requests in emergency department (ED). 

Second, all evaluations were at the referral to emergency department within the first hours of trauma; while we did not ‎assess patient-related delay in referral to ED and head trauma time to ED door remains a ‎confounding factor. ‎

In a 2013 review of 15 articles on salivary alpha-amylase as an indicator of autonomic nervous system dysregulation in healthy patients, it was found that salivary amylase was highly sensitive to stress-related changes, and salivary amylase could be an indicator of dysregulation of the autonomic nervous system in mental disorders [16]. It also indicates that previous anxiety disorders may be a confounding variable that should be assessed in further researches.

## Declarations

### Ethics approval and consent to participate:

The institutional review board (IRB) of Jahrom University of Medical Sciences (MUMS) approved this study with Code of IR.MUMS.MEDICAL.REC.1397.546 Respecting the patients’ autonomy, no identifiable information was reported.

### Consent for publication:

None declared.

### Conflict of interests:

There are no conflicts of interest.

### Funding:

None. 

### Authors’ contributions:

All the authors met the criteria of authorship based on the recommendations of the international Committee of Medical Journal Editors.

### Acknowledgements:

We would like to thank the Clinical Research Development Unit of Peymanieh Educational and Research and Therapeutic Center of Jahrom University of Medical Sciences for edit manuscript
